# Determination of Glyphosate in Water from a Rural Locality in México and Its Implications for the Population Based on Water Consumption and Use Habits

**DOI:** 10.3390/ijerph17197102

**Published:** 2020-09-28

**Authors:** Eduardo C. Reynoso, Ricardo D. Peña, Delfino Reyes, Yaselda Chavarin-Pineda, Ilaria Palchetti, Eduardo Torres

**Affiliations:** 1 Posgrado en Ciencias Ambientales, Instituto de Ciencias, Benemérita, Universidad Autónoma de Puebla, Puebla 72570, Mexico; eduardoc.reynoso@gmail.com (E.C.R.); yaschp@hotmail.com (Y.C.-P.); 2 Centro de Quìmica, Benemérita Universidad Autónoma de Puebla, Puebla 72570, Mexico; ricardo.pena@correo.buap.mx; 3 Facultad de Ingeniería Agrohidráulica, Benemérita Universidad Autónoma de Puebla, Av. Universidad s/n, Teziutlán, Puebla 73695, Mexico; delfino.reyes@correo.buap.mx; 4 Dipartimento di Chimica, Università degli Studi di Firenze, Via della Lastruccia 3, 50019 Sesto Fiorentino, Italy; ilaria.palchetti@unifi.it

**Keywords:** glyphosate, human exposure, water pollution

## Abstract

Glyphosate is a broad-spectrum herbicide widely used worldwide. Indeed, it is the herbicide most applied to all Mexican crops. Due to the overuse and poor disposal of the waste, this herbicide can reach the aquatic environments such as groundwater and surface water. Thus, there is a clear need to implement monitoring and surveillance programs for evaluating and controlling the exposure to this herbicide in rural populations. The goal of this study was to quantify the presence of glyphosate in different water bodies (groundwater, surface and drinking water) as well as to identify the uses and managements of water resources by rural communities to evaluate the potential human exposure to glyphosate in the Tenampulco region of the Mexican state of Puebla. Measurements were performed by a rapid and cost-effective ELISA-based method in groundwater and surface water from various sampling sites of the Tenampulco region. Glyphosate was detected in all groundwater samples to be below the maximum limit for glyphosate in water in Mexico. Nevertheless, these results indicate an exposure of glyphosate in these agricultural communities and the need to establish a monitoring program.

## 1. Introduction

As part of the systemic and broad-spectrum herbicides, glyphosate has the place of honor as the best-selling and most widely used herbicide globally [1]. It is estimated that over 800 million kilograms are sold annually with a value of over $6.5 billions dollars and it is usually applied on average 2–4 kg/ha [2], mainly in genetically modified crops resistant to this pesticide, such as soybeans, cotton and corn, among others, to eliminate unwanted herbs; their use is also common in native crops and gardening [3].

Glyphosate is usually applied by spraying [4], so that a high percentage is deposited directly on the soil [5,6]. Dispersion to other environmental compartments (water, plants and atmosphere) and the final destination will depend mainly on its interaction with the soil components [6]. 

Due to the extensive use and poor disposal of the waste, this herbicide is widely distributed in aquatic environments such as groundwater and surface water around the world; trace amounts have been reported in Australia (0.0011 mg/L) [1], Canada (0.0006 mg/L) [7], France (0.0059 mg/L) [8] and Argentina (0.002 mg/L) [9]. In Mexico, it has been reported for two cities, Campeche (0.0017 mg/L) [10] and Chiapas (0.0184 mg/L) [11]. 

Glyphosate is currently listed by the World Health Organization (WHO) in group 2A as a possible human carcinogen [12]. Regarding human exposure, the presence of glyphosate in human biological matrices is largely due to occupational exposure and trace consumption of contaminated food and water [13]. It has been found in human urine at concentrations between 0.4 and 18 μg/L with a half-life of 6 h [10,13,14,15]. 

**Figure 1:**
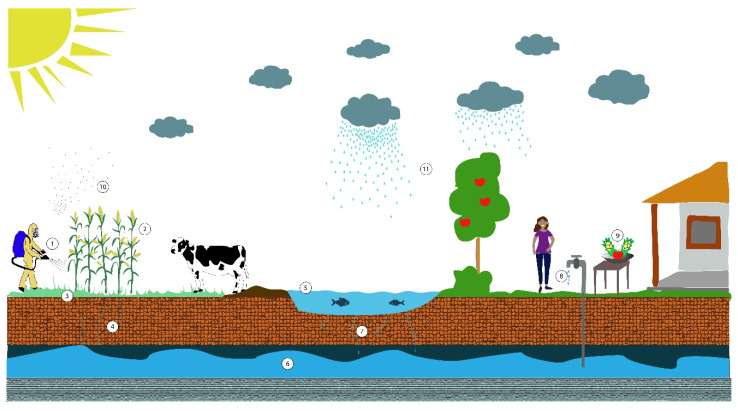
Human exposure routes to glyphosate. From the application of glyphosate (1) in plants (2), it can pass to different environments and expose the population through intake of contaminated food plants and by animals for human consumption that eat plants with glyphosate. A proportion of applied glyphosate reaches the soil (3) and through runoff and leaching (4) can reach surface water (5), such as rivers and lakes, and groundwater (6). Surface water (5) is used to irrigate crops (2), feed livestock and household activities, thus exposure is through animal and plant consumption and by ingestion and dermal absorption. By infiltration (7), contaminated surface water reaches the groundwater (6), which exposes people by drinking it (8), consumption of food washed with water (9) and dermal absorption by bathing. Finally, glyphosate aerosols (10) may also exert potential health risk through inhalation or dermal absorption after precipitation (11) or sedimentation.

From the concentration of glyphosate in dietary residues, environmental monitoring and biological fluids, such as urine or breast milk, it is possible to estimate the human exposure to pollutants present in the environment. The routes of exposure are different depending on the human activities and the environmental compartments (Figure 1); for example, in people who are not farmers, the main route of exposure is through the consumption of contaminated food and water. Stephenson and Harris (2016) evaluated glyphosate exposure from theoretical maximum daily intake (TMDI) in the diet using probabilistic and deterministic methods of maximum residue level (MRL), finding values well below the acceptable daily intake ADI [16]. In 2019, the EPA proposed guidance to assess human exposure to chemicals in a non-occupational setting from inhalation, ingestion and dermal exposure in terms of site characteristics, population and period time [17].

In drinking water, the maximum legally permitted limit depends on the legislation of each country. It is 280 μg/L in Canada and Argentina, 1 mg/L in Australia, 0.1 μg/L in the European Union and 700 μg/L in the United States of America (USA) and China [18]. This disparity among the different countries is due to the legislation on the use of GMOs and also due to the number of hectares of soil where this herbicide is applied [19]. In Mexico, the Federal Commission for Protection Against Sanitary Risks (COFEPRIS) agrees with the EPA and the European Commission, since it classifies glyphosate in group 4 as slightly toxic and non-carcinogenic and the maximum limit allowed in drinking water is 10 μg/L [20]. Despite the numerous studies on the detection and impact of glyphosate at the international level, at the national level, specifically in Mexico, more information is necessary for a complete diagnosis of the contamination of glyphosate. The diagnosis of the presence of the herbicide in the bodies of water, as well as the habits of use of the pesticide and water consumption of the population, will allow, eventually, to outline policies and implement programs to minimize environmental and human risk. The goal of this study was to quantify the presence of glyphosate in different water bodies (surface, groundwater and bottled drinking water) as well as to identify the uses and management of water by the community inhabitants to evaluate the potential human exposure in a rural locality of Mexico.

## 2. Materials and Methods

### 2.1. Study Site Description, Water Sample Collection and Physicochemical Water Characterization

**Figure 2:**
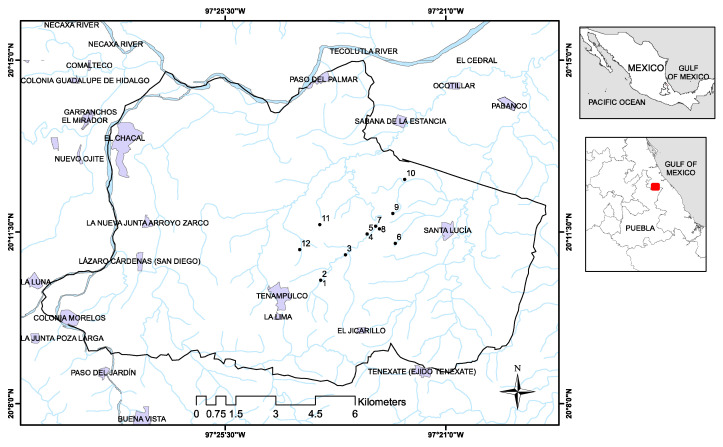
Geolocation of Tenampulco and sampling sites.

The municipality of Tenampulco is located in Region II of the northeastern highlands of the State of Puebla, Mexico (Figure 2). Its coordinates are 20°10′16″ N, 97°24′19″ W. It has an area of 140 km^2^ and, according to the National Institute of Statistics and Geography (INEGI), the population was 6975 in 2015 [21]. The climate is warm-humid, with an average annual temperature of more than 24 °C and rainfall of more than 2000 mm per year. The typical vegetation in the locality is high evergreen forest. In Tenampulco, the following land uses and vegetation are registered (percentage of the total territory): agriculture (44%, of which half is subsistence farming—corn—and the other half is commercial agriculture—citrus), urban area (0.5%), grassland (50%), jungle (3%) and forest (2%) [21].

Forty-eight samples of water (groundwater, surface and drinking bottled water) were obtained from different locations in Tenampulco (Figure 2) (Table 1) during four sampling campaigns in different periods of time (twelve samples each period): Period 1 (May 2018), Period 2 (August 2018), Period 3 (January 2019) and Period 4 (November 2019). The samples were collected from groundwater used for human consumption and agricultural purposes. The samples were collected from surface water bodies not used for human consumption, but they are used for irrigation of crops. The bottled drinking water (Sample 2) is commercialized for human consumption after physicochemical treatment of groundwater collected from Sampling Site 1.

Each water sample was characterized directly at the collection point for pH, conductivity and temperature (Conductronic, Puebla, México).

The water samples were collected, transported and stored at 4 °C in 200-mL amber glass bottles until their analysis. A photometric method based on chemical water quality test kit (Hanna Instruments, Woonsocket, RI, USA) was used for the physicochemical characterization of the water samples; in particular, reagents were used for the determination of phosphate (HI 93713-01), sulfate (Hl 93751-01), iron (Hl 93746-01), calcium (Hl 937521-01), nitrate (Hl 93728-01), free chlorine (Hl 93701-01), magnesium (Hl 937520-01) and chemical oxygen demand (COD) (HI 93754C-25). The biochemical oxygen demand (BOD) was determined according to the OECD 301 “guideline for testing of chemicals” [22] and the maximum levels described in the NOM-003-ECOL-1997 standard [23].

### 2.2. Determination of Glyphosate

Glyphosate determination was performed by using an ELISA kit following the procedure established by the manufacturer (Abraxis, Warminster, PA, USA) for determining glyphosate in water (PN 500081) [24]. The kit was validated by comparison to HPLC-based method [25] and no statistically significant differences (95% confidence interval) were found between the ELISA and HPLC analysis of the three water matrixes (Nanopure, tap and river waters). Thus, the ELISA method was used in this study as a rapid, cost-effective and reliable method for monitoring of glyphosate concentration in different water samples. The technique is based on a competitive assay, where glyphosate is indirectly measured by the quantification of color change produced by the enzymatic oxidation of 3,3’,5,5’-tetramethylbenzidine [19,26]. The change in absorbance at 450 nm was measured using a spectrophotometer Varian Cary 50, (Varian, Palo Alto, CA, USA). The detection limit and maximum detected established by the manufacturer are 0.05 and 4 μg/L respectively. A calibration curve was prepared for each of the four samples with concentrations ranging from 0.075 to 4 μg/L. For the determination of the glyphosate content in the samples, each sample was first filtered through a 0.4-µm cellulose filter; later, a fixed volume of 20 µL was added to the derivatized mixture of the detection kit. Then, 250 µL of the derivatized samples were mixed with 250 µL of enzyme conjugate and 500 µL of diluted anti-glyphosate particles and incubated at 25 °C for 30 min. The reaction mixture was separated using the magnetic separator and washed twice with 1 mL of distilled water. The TMB oxidation was carried out for 20 min at 25 °C and stopped by the addition of 500 µL of 0.5% sulfuric acid [24]. The reported values are the mean of two replicates.

### 2.3. Social Evaluation

To identify the factors associated with contamination of water with glyphosate, as well as to assess the potential human exposure of the inhabitants of Tenampulco, 38 face-to-face interviews based on structured questionnaires were used. The interviews recorded different aspects of herbicide use, with particular emphasis on farmers and their exposure to glyphosate and the dynamics of water consumption and use in the population near the sampling points to estimate the actual amounts of glyphosate consumption.

The people were chosen for convenience through non-probabilistic sampling to key people in the municipality who work and/or live near the sampling points; these people may or may not be farmers. It is important to mention that the decisions of the inhabitants of the locality are carried out in community meetings. A meeting was then held with the local inhabitants to explain the project and the objectives of the work. During the meeting, they were asked their availability to be interviewed on aspects of the use of drinking water in their daily work. All inhabitants agreed to allow us to carry out the analyses, visits and interviews. The questionaries were clearly explained to interviewees before starting; the interviewees were supplied with a copy of the written version of the same. The interviewees were informed about the confidentiality aspects; no name is placed on the final report. All the interviewees agreed to be recorded in a written report. 

### 2.4. Exposure Potential

**Figure 3:**
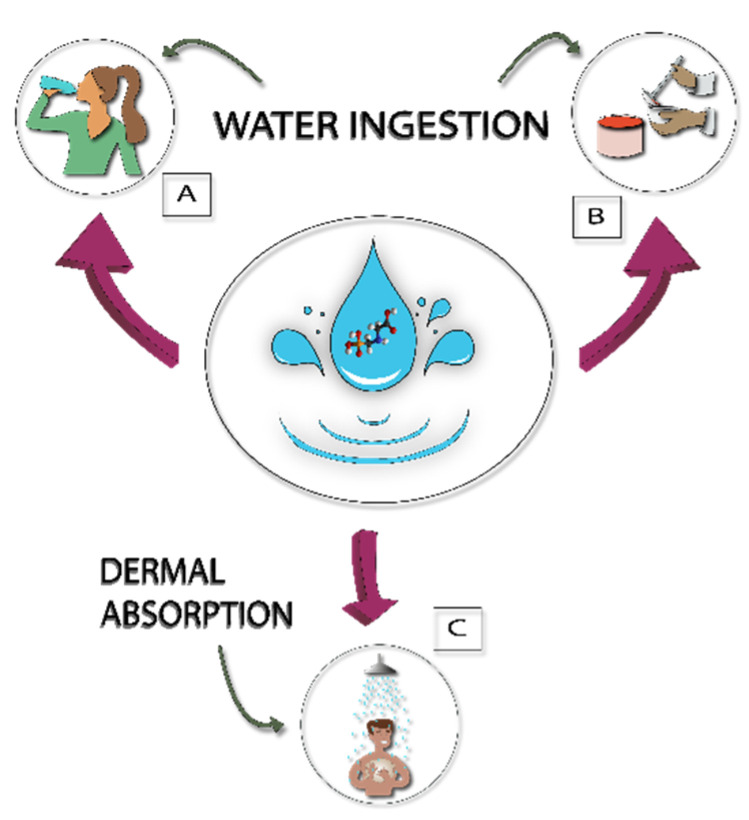
Social activities that affect human exposure to glyphosate from polluted water: (**A**) drinking water; (**B**) food preparation; and (**C**) showering.

Human exposure to glyphosate herbicide is a complex problem that is linked to different factors, both environmental and social, as shown in Figure 1 [27]. In this work, the approach evaluated the exposure to glyphosate from polluted water used for consumption and household activities (Figure 3).

To estimate the potential glyphosate exposure, the glyphosate concentration from each sampling site was multiplied by the amount of water consumed. The amounts of water consumed was estimated according to the interviews with key information; in this way, the variables were (Figure 3): *amount of glyphosate ingested by drinking water* (i.e., the average amount of glyphosate consumed directly by water from wells); *amount of glyphosate consumed for food preparation*; and *amount of glyphosate absorbed by the skin by water during showering*. Glyphosate can be absorbed by the skin from direct contact with water contaminated with the pesticide. A percentage of 0.8%, proposed by Westes et al. [28], was used in this study; this means that if a person uses 20 L of water for bathing at a concentration of 1 g/L, the amount absorbed by the skin will be 0.16 g/L. The potential exposure for each sampling site was calculated as though the people consumed water for all their needs in that sampling point.

## 3. Results and Discussions

### 3.1. Study Site Results, Water Sample Collection and Water Characterization

Tenampulco has an average height of 163 m above sea level (range from 93 to 1210 m), rainfall exceeding 1900 mm per year (monthly value from 53 to 627 mm) and an average temperature of 31 °C (range from 22 to 35 °C), which makes it an ideal climate and altitude for the cultivation of vanilla and citrus fruits such as tangerine, orange or grapefruit; despite the presence of slopes, the place is also used for the cultivation of corn, the main crop planted in the region. The geographic features of the region favor the dispersion of pollutants from the sources to the rest of the locality, especially to those places where water is destined for human use. This makes the population vulnerable to ingest glyphosate and other agrochemicals in a non-voluntary way.

**Figure 4:**
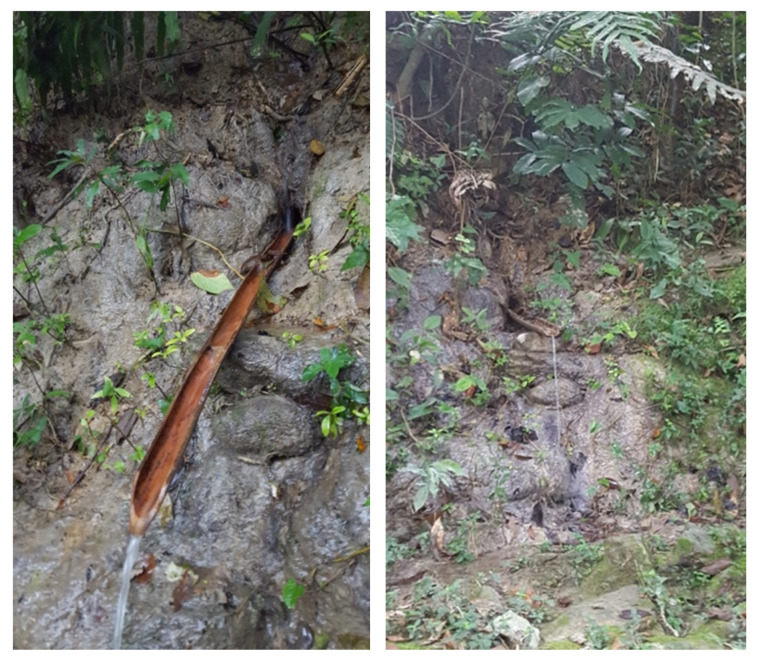
Sampling Point 6 runoff water.

**Figure 5:**
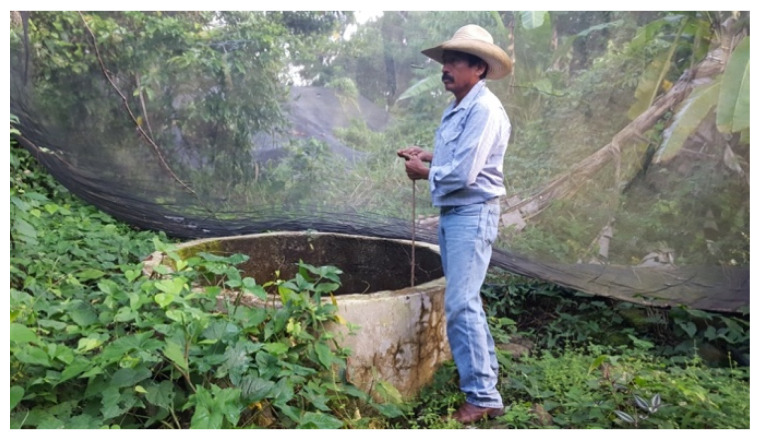
Sample Point 9 well.

All sampling points were selected to assess the presence of glyphosate as well as its relationship with the economic or social activity carried out around these points. Point 1 is the leading well where water is extracted for the purification treatment site (Point 2), and then distributed in some parts of the town. Points 3, 4, 5, 7 and 10 are surface water basins that receive runoff from nearby crop fields. Point 6 is runoff water from the infiltration of agricultural fields (Figure 4). Points 8, 9 and 12 are wells used by the population for consumption and other household activities (Figure 5). Finally, point 11 is stagnant water, where the population washes and dispose of empty containers containing residual pesticide formulations.

The average water temperature was 24 °C (range from 18 to 28.2 °C); it is essential to note that the samples were taken between 7:00 and 10:00. The average conductivity was 595 μs/cm (range from 468 to 697 μs/cm), which is considered suitable for irrigation since it presents values below 750 μs/cm [29]. According to the permissible limits for pH in water in Mexico (6.5–8.5), all values were within this limit (average 7.4, range from 6.8 to 8.2) [30]. The values of these physicochemical parameters allow the persistence of glyphosate in the ecosystem, given the stability of the molecule [31].

The description of the next parameters is based on the limits established by the Mexican regulations. Table 2 summarizes the global values of all samples analyzed. Sulfate and free chlorine have a maximum limit in the water of 400 and 1.5 mg/L, respectively [30]; none of the evaluated samples exceeded these values. Nitrate is an anion that is found naturally dissolved in water and its presence is due to the nitrogen cycle. In groundwater and surface waters without human activities, nitrate should not exceed 2 mg/L; higher concentrations of nitrates are mainly due to use of agricultural fertilizers, manure and compost fertilizers [32,33]. Sampling Points 1, 6 and 9 showed a high concentration of nitrates (13.9, 19.7 and 57 mg/L respectively) due to the proximity of crops and livestock raising. The Ministry of Health establishes a maximum limit for nitrates of 10 mg/L [30].

As glyphosate is a chelating compound, it can divalently bind iron, calcium and magnesium forming stable complexes, therefore the presence of iron in the soil or water can cause glyphosate to persist for several months [34]. In Tenampulco, although below the national permissible limits, significant amounts of cations were observed in all samples, with values of 77.1 µg/L for iron, 141 mg/L for calcium and 8.1 mg/L and for magnesium [30]. On the other side, anions can bind particle soils, avoiding glyphosate being immobilized in the soil, and then it might be lixiviated easily. In this sense, the concentration of phosphate was 0.5 mg/L, which is low for avoiding glyphosate adsorption on soil particles [35].

Finally, the COD indicates the amount of inorganic and organic matter, whether biodegradable or not; an increase in this value indicates the presence of substances from non-municipal discharges or the presence of recalcitrant compounds. According to the national legislation [36], COD values indicated that the water from Tenampulco is classified as contaminated (40–200 mg/L), and in some cases (Points 9 and 11) it is considered heavily contaminated (more than 200 mg/L). According to the BOD_5_ values, it is established that the contaminants present in the water are mainly non-biodegradable since no values were found that indicate biodegradable contaminants.

### 3.2. Glyphosate Detection

Glyphosate was detected in all the sampling periods, even during the spring and summer seasons, when abundant rainfall takes place (Table 3); in none of the cases, the glyphosate level was above the maximum limit for glyphosate in water for Mexico (10 µg/L). In the sowing periods (summer and winter), higher glyphosate concentrations were observed. Sampling Sites 2, 4 and 5 did not show the presence of the herbicide; as expected, Site 2, the purification treatment site, was good enough for glyphosate removal, while Points 4 and 5, which correspond to surface water basins, were also expected to be characterized by the absence of the herbicide. Sampling Sites 1, 6, 8, 9 and 12 are of the most concern because these are for use and consumption by the population, and, in all cases, was the presence of glyphosate in at least two seasons. Sampling Site 11 presented the highest concentrations because it is used for washing and discarding empty pesticide containers. Sampling Sites 3, 7 and 10 are river waters; glyphosate in these sites is due to the crops near the sampling areas and small streams that flow into the river.

In Mexico, there are two reports of the presence of glyphosate in water. In Campeche, Rendón and Dzul (2017) analyzed samples of groundwater, bottled drinking water and urine from the population [10]. In their study, the presence of the herbicide was found in 90% of the 29 ground water samples analyzed, with a maximum concentration of 1.58 µg/L and an average of 1.05 µg/L [10]. In our investigation, 100% of the groundwater samples from Periods 3 and 4 showed the presence of the herbicide with an average value of almost 1.18 µg/L. In comparison to the previous study, it is essential to note that the Campeche region is characterized by an intensive use of genetically modified organisms resistant to glyphosate, mainly soybeans and cotton [37], so that the use of glyphosate in crops is high. In Chiapas, Ruiz-Toledo and collaborators (2014) evaluated the presence of glyphosate in two periods of time (February and July) for different water bodies (surface and underground). The results of their study showed that in the dry season there is a higher glyphosate concentration (average and maximum concentration of 5.69 and 36.71 µg/L, respectively) than in the rainy season (average and maximum concentration of 0.35 and 1.33 µg/L, respectively) [11]. The same trend was observed in our research since the higher concentrations of glyphosate were determined in the dry season (autumn and winter, average and maximum concentration of 1.56 and 4.33 µg/L, respectively), than in rainy seasons (spring and summer average and maximum concentration 0.87 and 4.36 µg/L, respectively); the disparity of the values found is mainly due to its continuous application on a great diversity of crops in Chiapas area [11].

At the international level, in recent years the presence of glyphosate has been found in both ground and surface water in Canada [38], Argentina [39] and Sri Lanka [40]. The values found are very similar to was found in Tenampulco, except in Argentina, due to the excessive use of glyphosate on crops where genetically modified organisms are planted [41]. Although the main route of passage of glyphosate to water is by agricultural activity, a fraction is due to home activities and gardening [42].

### 3.3. Social Evaluation

By the enrollment of key people of the municipality, verbal interviews to 38 participants were collected, in order to learn about the use and management of glyphosate and water resources. The participants consisted of 55% women and 45% men from 14 to 74 of age. The two main activities of the population were agriculture (47%) and household (53%).

**Figure 6:**
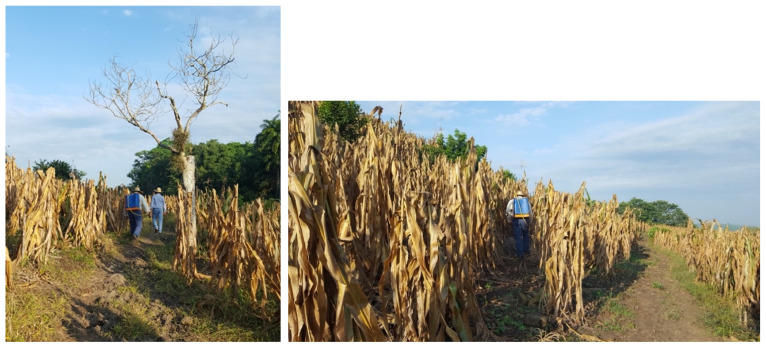
Farmer applying glyphosate with backpack sprayer on corn crops.

The first section of the interview focused on how farmers manage their crops and the use of pesticides. Maize is the main crop in the region, as 94% of the farmers grow it; the remaining 6% grow only citrus fruits such as oranges and tangerines. Most of the people grow around 5 ha, though some of them grow the maximum of 10 ha. Grass or Weeds are the main pest and glyphosate is the primary pesticide used by 100% of farmers and 72% combine it with 2,4-D (2,4-dichlorophenoxyacetic acid) and paraquat. A critical aspect was observed regarding the safety conditions, more than half of the farmers interviewed (56%) do not use any equipment or safety measures, just a simple handkerchief or bandana (Figure 6). This is a concern because the label on the packaging states the use of masks, coveralls, gloves and rubber boots because of the well-known toxicity of some pesticides [43].

Another critical issue is the disposal of empty pesticide containers because, despite the existence of programs for washing and collecting empty pesticide containers in the country [44], 50% of people in Tenampulco burn in the open, 22% throw away the containers and nobody disposes of them of in special containers, because the collection program does not reach the municipality.

For the application of glyphosate, the farmers use the spray backpack to apply the herbicide to the unwanted plants (Figure 6), mainly in the winter and summer season, before cultivation. The initial concentration used by operators to prepare the working solution is 41% glyphosate in the form of isopropylamine salt (360 g of glyphosate per liter of solution). Generally, to prepare the spray solution, 100–200 mL of concentrated glyphosate is poured in 20 L of water (backpack capacity); 67% of farmers use ten backpacks (360 g of glyphosate) per hectare of the crop, the remaining 33% use 720 g of glyphosate per hectare. Regarding the frequency of application, 11% of farmers apply glyphosate once a month, 17% once every 15 days, 17% apply 2–3 times a week, 22% apply once a week and the highest percentage number were those who apply daily (28%). All farmers mix glyphosate with other pesticides and herbicides, mainly 2,4-D and paraquat; the exposure of these two pesticides, along with glyphosate, is of great importance and generates more significant interest in studying their presence and distribution because of the toxicity and public health implications they produce, since exposure to Paraquat and 2,4-D has been reported to represent a risk in Parkinson’s disease and non-Hodgkin’s lymphoma, respectively [45,46]. Moreover, 50% of them mentioned the necessity of these mixtures because of the increasing resistance of weeds.

**Figure 7:**
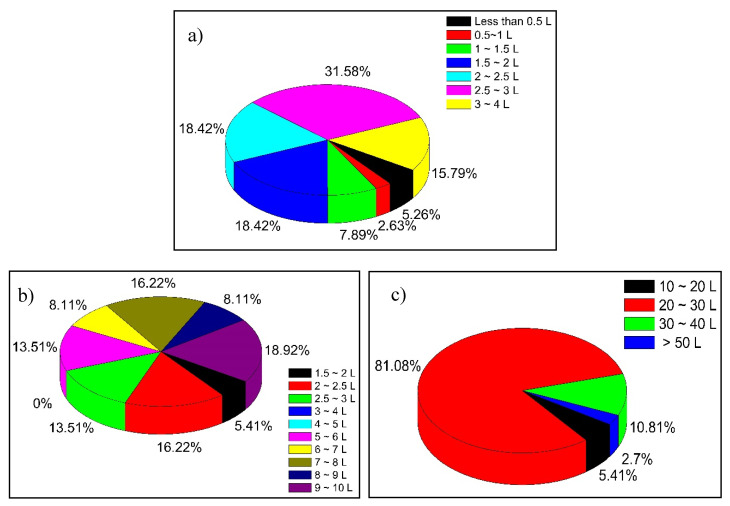
Amount of water, expressed in liters per day, used by the inhabitants for: (**a**) drinking; (**b**) preparing food; and (**c**) bathing.

Finally, the use and consumption of water was addressed. According to the interviews, 80% of the population obtains water directly from wells, 16% obtain it from municipal piped water and the rest buy bottled water. The high temperature of the region throughout the year favors water consumption; two-thirds of the population consumes more than 2 L of water per day (Figure 7a). For food preparation, 63% of the population consumes more than 5 L of water (Figure 7b). Finally, the amount of water used by the population for bathing was evaluated, since a percentage of glyphosate contained in the water can be absorbed through the skin. Figure 7c shows that the majority of the population (81%) uses between 20 and 30 L of water for bathing. These last three parameters were necessary to estimate the amount of glyphosate ingested by the population according to the uses and patterns of water consumption.

### 3.4. Potential Glyphosate Exposure 

**Figure 8:**
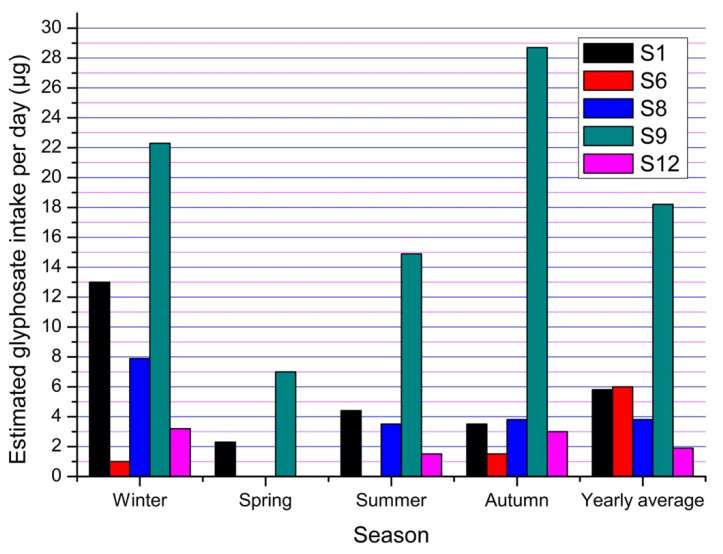
Estimation of the amount of glyphosate intake per day according to water use and consumption habits at Sampling Sites 1, 6, 8, 9 and 12.

The average daily drinking water consumption per person was 2.6 L and the amount of water used to prepare food was 6.2 L. In addition, 31 L per day is the average used by a person in Tenampulco to bathe. With these data, the average potential exposure values were obtained for the most representative Sampling Sites 1, 6, 8, 9 and 12 (Figure 8) for the different seasons of analysis, as well as for the whole year.

As shown in Figure 8, winter and autumn are the periods in which the population ingests the most glyphosate. Sampling Site 9 has the highest concentration of glyphosate during the year and has the highest level of potential exposure to the population, although the water from this well (Figure 5) is used mainly to irrigate the crops in the area (citrus fruits, corn and vanilla) and farmers do not regularly consume it. Sampling Sites 8 and 12 are in direct connection to all taps in nearby houses; hence, the amount of glyphosate calculated gives a closer estimate of human exposure.

Sampling Site 6 has the lowest potential exposure levels compared to the other four sites despite being runoff water, which normally dissolves the deposited herbicide in soil; the water from this site does not have the destination of any household as it flows directly into the river; however, it represents a potential problem because people living near the site drink from this water during workday.

The average amount of glyphosate that the population of Tenampulco is potentially exposed to through water use and consumption from wells is 6.1 µg per day, although there are places where the amount of glyphosate exceeds 20 µg per day. Exposure values can be significantly increased by diet [16] and skin absorption from the atmosphere [47].

Figure 8 shows the amount of glyphosate that the population of Tenampulco is likely to be exposed to in a year. Factors such as rainfall [11], orography and hydrogeology [9] of the region favor that, in some spots of the municipality and during the different seasons of the year, there is a greater probability of ingesting the herbicide.

Glyphosate is mainly used in corn crops and is later transported to other reservoirs, mainly wells, where it will be used by the population [31], and groundwater. Due to low rainfall conditions, it can persist for several months [48], as observed in winter. Several authors have reported similar amounts of glyphosate to those determined in this study [7,10], but most evaluate the concentrations based only on water, soil, air or food [49], without taking into account the management of the population; with these population indicators, it is possible to have a more realistic approximation of the exposure of humans and other organisms to glyphosate.

It should be noted that the concentrations found can alter the different ecosystems, causing loss of biodiversity and exposure to specific organisms, triggering significant problems in the decomposition and cycling of nutrients, such as soil microorganisms, fish, worms and even birds and insects, such as bees [42,50]. When passed into other environmental compartments such as the air, glyphosate can cause lung diseases, such as asthma in humans [51].

Due to increasing use around the world and epidemiological studies in humans and animals, it has been suggested by several authors that glyphosate exposure is related to diseases such as breast and prostate cancer, genotoxicity and oxidative stress [52]. For this reason, several institutions have emphasized monitoring the presence of glyphosate, not only in the environment but also in organisms that help to measure the increase in its presence and the various alterations produced [53].

## 4. Conclusions

According to Mexican regulations, none of the samples exceed the maximum permissible limit for glyphosate in water. However, according to the different water management activities in the population of Tenampulco, the accumulated consumption of glyphosate in some sites exceeds 20 μg per day. The amount of glyphosate that can be potentially ingested by a person through different exposure routes might be significantly increased by food and atmospheric pathways.

The weather and conditions, the crop cycles and application of glyphosate can significantly influence the levels to which the population may be exposed, because average temperature and rainfall in Tenampulco favor the presence of the herbicide, especially in the winter season when lower temperatures and less rain are the prevalent conditions.

Agricultural practices influence the contamination of the water resource, as all samples are contaminated by recalcitrant compounds that are not easily degradable and that diminish their quality. The handling and use of agrochemicals are not optimal since safety measures and applications by agricultural workers are deficient and, in many cases, do not meet the minimum specifications recommended by the producer.

The lack of attention and responsibility of the authorities to disseminate the consequences of the misuse of pesticides and the absence of management plans for pesticides and their residues favors the presence of the herbicide in the region; therefore, it is necessary to implement monitoring and surveillance programs to evaluate, prevent and control exposure to herbicides in the population and the environment.

It is necessary to carry out studies to estimate exposure through contact with the atmosphere and food, such as animals for human consumption and seasonal food in the region, in order to determine the risk to human health and ecotoxicology.

## Figures and Tables

**Table 1:** Coordinates and type of water collected at the different sampling points.

Sample Number	Water Type	Coordinates	
		Latitude N	Longitude W
1	Groundwater	20°10′31.12″	97°23′33.22″
2	Bottled water	20°10′31.16″	97°23′33.18″
3	Surface water	20°10′37.22″	97°23′22.68″
4	Surface water	20°11′27.80″	97°22′36.44″
5	Surface water	20°11′27.79″	97°22′36.43″
6	Runoff water	20°11′16.33″	97°22′01.99″
7	Surface water	20°11′33.95″	97°22′21.17″
8	Groundwater	20°11′37.44″	97°22′25.95″
9	Groundwater	20°11′52.99″	97°22′04.99″
10	Surface water	20°12′34.44″	97°21′50.32″
11	Surface water	20°11′39.37″	97°23′34.19″
12	Groundwater	20°11′08.79″	97°23′58.87″

**Table 2:** Descriptive statistics of the water quality values of all sampling points.

Parameter	Phosphate (mg/L)	Sulfate (mg/L)	Iron (μg/L)	Nitrate (mg/L)	Free Chlorine (mg/L)	Calcium (mg/L)	Magnesium (mg/L)	COD * (mg/L)	BOD ** (mg/L)
Mean	0.5	15.9	77.1	5.5	0.05	141	8.1	167	0.26
SD ***	0.2	5.7	45.8	8.8	0.03	32	7.9	103	0.16
Maximum	1.1	30.0	165.0	27.7	0.13	220	35.0	483	0.70
Minimum	0.0	0.0	6.0	0.0	0.01	80	0.0	37	0.05

* COD, chemical oxygen demand; ** BOD, biochemical oxygen demand; *** SD, standard deviation;

**Table 3:** Average glyphosate concentrations in the water samples during the four time periods.

Sample Point	Concentration 1st Sampling (µg/L) (Spring)	Concentration 2nd Sampling (µg/L) (Summer)	Concentration 3rd Sampling (µg/L) (Winter)	Concentration 4th Sampling (µg/L) (Autumn)
1	0.26 ± 0.01	0.49 ± 0.03	1.43 ± 0.01	0.38 ± 0.02
2	<LOD	<LOD	<LOD	<LOD
3	0.37 ± 0.03	1.11 ± 0.10	2.59 ± 0.14	1.55 ± 0.11
4	<LOD	<LOD	<LOD	<LOD
5	<LOD	<LOD	<LOD	<LOD
6	<LOD	<LOD	0.11 ± 0.01	0.17 ± 0.02
7	0.33 ± 0.02	0.52 ± 0.07	1.58 ± 0.08	3.28 ± 0.10
8	<LOD	0.39 ± 0.02	0.88 ± 0.03	0.42 ± 0.04
9	0.77 ± 0.06	1.64 ± 0.18	2.46 ± 0.24	3.17 ± 0.33
10	0.53 ± 0.03	0.48 ± 0.031	1.31 ± 0.15	0.58 ± 0.03
11	0.81 ± 0.04	4.36 ± 0.20	3.11 ± 0.26	4.33 ± 0.35
12	<LOD	0.16 ± 0.02	0.36 ± 0.05	0.33 ± 0.40

LOD, Limit of Detection.
